# 4-Aminobutyrate Aminotransferase (ABAT): Genetic and Pharmacological Evidence for an Involvement in Gastro Esophageal Reflux Disease

**DOI:** 10.1371/journal.pone.0019095

**Published:** 2011-04-28

**Authors:** Johan Jirholt, Bengt Åsling, Paul Hammond, Geoffrey Davidson, Mikael Knutsson, Anna Walentinsson, Jörgen M. Jensen, Anders Lehmann, Lars Agreus, Maria Lagerström-Fermer

**Affiliations:** 1 AstraZeneca R&D, Mölndal, Sweden; 2 Gastroenterology Unit, Women's & Children's Hospital, North Adelaide, Australia; 3 Center for Family and Community Medicine, Karolinska Institutet, Huddinge, Sweden; Universite de Montreal, Canada

## Abstract

Gastro-esophageal reflux disease (GERD) is partly caused by genetic factors. The underlying susceptibility genes are currently unknown, with the exception of COL3A1. We used three independent GERD patient cohorts to identify GERD susceptibility genes. Thirty-six families, demonstrating dominant transmission of GERD were subjected to whole genome microsatellite genotyping and linkage analysis. Five linked regions were identified. Two families shared a linked region (LOD 3.9 and 2.0) on chromosome 16. We used two additional independent GERD patient cohorts, one consisting of 219 trios (affected child with parents) and the other an adult GERD case control cohort consisting of 256 cases and 485 controls, to validate individual genes in the linked region through association analysis. Sixty six single nucleotide polymorphism (SNP) markers distributed over the nine genes present in the linked region were genotyped in the independent GERD trio cohort. Transmission disequilibrium test analysis followed by multiple testing adjustments revealed a significant genetic association for one SNP located in an intron of the gene 4-aminobutyrate aminotransferase (ABAT) (P_adj_ = 0.027). This association did not replicate in the adult case-control cohort, possibly due to the differences in ethnicity between the cohorts. Finally, using the selective ABAT inhibitor vigabatrin (γ-vinyl GABA) in a dog study, we were able to show a reduction of transient lower esophageal sphincter relaxations (TLESRs) by 57.3±11.4 % (p = 0.007) and the reflux events from 3.1±0.4 to 0.8±0.4 (p = 0.007). Our results demonstrate the direct involvement of ABAT in pathways affecting lower esophageal sphincter (LES) control and identifies ABAT as a genetic risk factor for GERD.

## Introduction

Gastroesophageal reflux disease (GERD) is an increasingly common gastrointestinal disorder with a prevalence of 25–40% in the western world [1], [2]. GERD symptoms, such as heartburn and regurgitation, leave affected individuals with a severely impaired quality of life and place a considerable health economic burden on society [3], [4].

Diagnosis relies on clinical assessment and multidimensional questionnaires combined with physical examinations such as endoscopic examination and 24 hour pH measurements of the esophagus. The disease has been subdivided into erosive reflux disease (ERD) and non-erosive reflux disease (NERD) [5]. The patient classification helps predict likelihood of response to proton pump inhibitors, ERD individuals exhibiting a better response rate than NERD individuals. ERD is thought to be caused mainly by acid reflux into the esophagus while NERD may include other factors such as weakly acidic or non-acidic reflux. Less than half of affected individuals have the erosive form of the disease where the esophageal mucosa is damaged by acidic reflux. The majority suffer from NERD, where affected individuals experience typical GERD symptoms without visible esophageal injuries [6].

Onset of the disease is variable and occurs among children as well as in adults. It has been suggested that GERD may originate in childhood [1], [7]–[10]. In fact, GERD is the most common esophageal disorder in children and 11% of infants are affected during their first year of life [11]. However, there are differences between pediatric and adult GERD such that the erosive form of the disease is rarely seen in the young [12].

Both environmental and genetic factors contribute to disease susceptibility. There is a clear genetic component to GERD, as has been shown in several studies through familial clustering and twin concordance rates [13]–[16]. In the large twin study by Cameron et al. (2002), the genetic heritability was estimated at 31%. However, with the one exception of COL3A1, the identities of the causative genes are presently unknown. COL3A1 encodes collagen type III alpha 1 providing molecular support for the notion that there is a connective tissue weakness component to GERD [17].

A number of lifestyle and environmental factors such as obesity and alcohol consumption has also been shown to be associated with an increased risk of acquiring the disease [2]. It is probable that some of these environmental factors are not present in young GERD patients, suggesting that the genetic component may be more penetrant in the young.

Treatment of GERD is primarily provided through proton pump inhibitors (PPIs) that reduce the acidity of the stomach's gastric acid. However, a large proportion of GERD patients do not benefit from this treatment leaving a large unmet need for new pharmacological interventions. A large effort has been devoted to developing drugs that inhibit transient lower esophageal sphincter relaxations (TLESRs), the major underlying mechanism for gastroesophageal reflux [18]–[20], aiming to reduce the number of reflux events in affected individuals. This research has to some extent been dependent on animal models, where dogs and ferrets are the best established models [21], [22]. Future anti-reflux drugs hold the hope of being effective for the treatment of patients who experience GERD symptoms despite being on PPIs [23].

Genetics can be used to address the underlying disease mechanisms and provide potential new targets for drug treatment as well as biomarkers for patient stratification. We have used a combination of human genetic approaches coupled with validation in the TLESR dog model to identify 4-aminobutyrate aminotransferase (ABAT) as a potential GERD susceptibility gene affecting control of the LES.

## Methods

### Ethics statement

Written informed consent was obtained before enrolment. All data and DNA/tissue samples were coded. Ethical approval was obtained for all patient collections and human research from the Women's and Children's Hospital (Adelaide) Research Ethics Committee with reference numbers REC1262/11/2004 & REC1340/6/2005.

The adult case control cohort was approved by the Swedish regional ethics review board in Umeå with reference number 321/03, diary nr 03-285.

All procedures involving laboratory animals were approved by the regional animal ethics review board in Gothenburg, Sweden with reference number 366-2005.

### Patient collections

Three groups of GERD patients have been used in this study. The first is a cohort of subjects of all ages in whom there is a convincing family history of GERD (referred to as “Families”). The second is a cohort of children with GERD without a strong family history of GERD, along with their parents (referred to as “Trios”). The third is a collection of adult individuals suffering from GERD together with healthy control individuals (referred to as “adult case control cohort”).

### Ethnicity

The family and trio cohorts were collected from the diverse multi-ethnic Australian population [24]. The “ethnic strength” describes how much a specific ethnicity contributes to a specific population. For the Australian population, it is as follows: approximately 70% Anglo-Celtic, 18% European (including 0.28% Swedish and 0.13% Finnish), 6% Asian, 1.5% Aboriginal/Torrens Strait Islander and 4.5% African, Jewish, Pacific and Indigenous American collectively. The adult case control cohort was collected from north-eastern Sweden. This population is a mixture of largely three ethnic groups where the Finnish and Swedish contributions are roughly equal with an approximately 10% Saami admixture [25].

### Families

Enrolment of patients was carried out in the Gastroenterology Unit at Women's and Children's Hospital (WCH, Adelaide, Australia), from 2001 to 2005 by identifying children (age up to 18 years) diagnosed with GERD and displaying at least one of the following criteria within the last five years: an abnormal finding from endoscopic examination or 24 hours esophageal pH test or having been subjected to a fundoplication. Probands were identified by examination of WCH patient records and by contacting the local GERD support association (FIRSA; Friends of Infant Reflux Support Association). Parents of the proband were contacted and the family was enrolled if a positive family history of GERD could be shown. Disease status was assessed via physician diagnosis based on results of previously performed investigations. Patients with a medical condition known to predispose to GERD were excluded.

### Trios

The trio cohort consisted of pediatric patients diagnosed with GERD between three months and 17 years of age without conditions predisposing to GERD, and their corresponding parents. The diagnosis of GERD was based on the following: a pediatric gastroenterologist's evaluation of symptoms to be consistent with GERD, in addition to either pH probe determined acid exposure consistent with GERD, endoscopic findings consistent with GERD and/or a definite and significant improvement in symptoms of GERD shortly after commencing anti-reflux treatment. Previous anti-reflux surgery was also considered as evidence of GERD. Patients were identified from databases of endoscopy, pH probe and outpatient diagnoses from the Gastroenterology Unit at WCH. All the probands that were endoscopically evaluated also had a histological examination of esophageal biopsies. Grading of esophageal injuries found at endoscopic examinations was based on the Tytgat classification system [26], the most appropriate endoscopic grading system for pediatric GERD [27]. The classification of the histological examinations of esophageal biopsies was based on Knuff & Leape [28], [29]. There is a poor correlation between endoscopic evaluation and histological examinations in GERD diagnosis [27]. We therefore used both methods as inclusion criteria; individuals with positive findings from either endoscopy or histology evaluation were included in the trio cohort.

Patients were excluded if they had been diagnosed with a condition predisposing to GERD, such as severe (non-ambulant) cerebral palsy, cystic fibrosis, major congenital abnormalities of the gastrointestinal tract (e.g. esophageal atresia/tracheo-esophageal fistula), and severe chronic lung disease (for example oral steroid dependent asthmatics and bronchopulmonary dysplasia). The total number of individuals meeting these criteria was 219. The average age was 6 ½ years and these patients had an average BMI of 18.2 kg/m^2^. The cohort consisted of 65% males. The probands meeting the inclusion criteria were endoscopically classified into grade 0 through 5 with the frequencies 20%, 58%, 16%, 4%, 1%, and 1%, respectively. Correspondingly, histopathological grading resulted in 22%, 44%, 30%, 3%, 1%, and 1% of patients classified with grades 0 through 5, respectively.

### Adult GERD case control cohort

The Kalixanda cohort is a collection of patients from northern Sweden where epidemiological factors related to GERD have been investigated [30], [31]. A careful description of this cohort has previously been published [31] including confounding factors. In brief, all individuals went through an endoscopic examination that together with a written questionnaire was used to diagnose GERD by professional gastroenterologists. Using the same criteria and the same physicians, we extended this cohort with 100 additional GERD patients from the same demographic area, resulting in the adult case control cohort used in this study. The cohort consists of 256 adult GERD cases (39% males) and 485 healthy control individuals (46% males). The median age was 56 and 54 years in the case and control groups respectively.

### Animals

Ten adult Labrador retrievers were used in the study. Cervical esophagostomies [32] were made. After recovery from surgery the dogs were accustomed to rest in a Pavlov stand. Before the experiments, the dogs were fasted for approximately 16–18 h, but with free access to water.

### Techniques

#### DNA extraction

DNA was extracted from blood or buccal swabs using Qiagen (Qiagen, Valencia, CA, USA) or GenomPhi (Amersham Biosciences AB, Uppsala, Sweden) according to manufacturer's protocol. In cases where DNA amount was limited, whole genomes were amplified by Molecular Staging Inc (Denver, CO, USA) through their Repli-g service.

#### Genotyping

Microsatellite genotyping was performed in 419 DNA samples using ABI Prism Linkage Mapping set v2.5 HD5 for DNA fragment analysis (Applied Biosystems, Foster City, CA, USA). Additional microsatellites were amplified using public primer sequences. The forward primer was ordered 5′-fluorophore labeled (Sigma-Genosys, Cambridge, United Kingdom) while the reverse primer was optimized [33]. Size fractioning of DNA fragments was done in ABI 3700 or 3730 DNA analyzers (Applied Biosystems). Alleles were called using the Genotyper v3.0 software (Applied Biosystems). The mean distance between adjacent markers was 4.32 centimorgan (SD ± 2.65, range 0–14.78). Single nucleotide polymorphism (SNP) genotyping was performed using the TaqMan assay (Applied Biosystems) and detection in ABI 7900HT (Applied Biosystems) according to manufacturers recommendations, apart from reducing the total reaction volume to 2.5 µl. All data were analyzed with Sequence Detection Systems © software v2.1 (Applied Biosystems).

#### Sequencing

Sequencing was performed from both directions using the Applied Biosystems 3700 or 3730 automated DNA sequencers (Applied Biosystems Inc) according to the manufacturer's recommendation. Sequence analysis was done using (Sequencher 4.6, Genecodes, Ann Arbor, MI, USA) and compared to the public genomic sequence (Ensembl release 49). Sequence differences were manually checked and remaining inconsistencies were resequenced.

#### Measurement of TLESR

The method applied for the studies have been described previously [34]. Briefly, the dogs were intubated with a water-perfused Dentsleeve multilumen assembly to record the gastric, LES and esophageal pressures. An antimony pH electrode was placed 3 cm above the LES for measurement of acid reflux episodes and a water-perfused catheter was placed in the hypopharynx to measure swallows. TLESRs were stimulated by gastric infusion of an acidified liquid nutrient (30 ml/kg; 100 ml/min) followed by air insufflation (500 ml/min) to maintain a gastric pressure of 10±1 mmHg during the experiment. The number of TLESRs was measured during a 45 min period starting from infusion of the liquid. TLESRs were defined as a rapid decrease in LES pressure (>1 mmHg/s), to a pressure <2 mmHg above gastric pressure and a duration >1 s, without any pharyngeal signal <2 s before onset. Reflux episodes were defined as a drop in pH>1 unit within 5 seconds with an average pH of <4 in the 15 seconds following nadir pH. Vigabatrin (Sabrilex®) was dissolved in sterile water and administered intragastrically (100 mg/kg; 0.5 ml/kg) 2 h before start of measurement or 2 h and 16 h (100 mg/kg at each occasion) before start of measurement.

### Statistics

#### Quality Control of familial genotype data

Mendelian inconsistencies were detected using PedCheck (v.1.1) [35]. Markers with a large number of errors were either reanalyzed or excluded. Individual erroneous genotypes were either rechecked or discarded. Hardy-Weinberg testing was performed in Pedstats (v.0.6.4) [36]. Finally, Merlin (v.0.10.2) [37] and, in the case of family 146, SimWalk2 (v.2.91) [38]–[40] were used to find unlikely genotypes. Although markers deviating from Hardy-Weinberg equilibrium and unlikely genotypes were not automatically discarded, care was taken to ensure that they did not influence the linkage peaks.

### Quality control of the trio cohort and the adult case control cohort genotype data

Mendelian inconsistencies in trio genotypes were detected using PedCheck (v.1.1). Hardy-Weinberg testing as well as calculation of descriptive statistics was performed in both Pedstats (v.0.6.4) and Haploview v.3.32 [41] for both the Trios and the adult case control cohort. Hardy-Weinberg testing was used as a tool to find SNPs to reanalyze rather than to discard SNPs from the association analysis. However, it was made sure that positive associations could not be explained by deviations from Hardy-Weinberg equilibrium.

### Linkage analysis

Genetic map positions used in the linkage analysis were obtained from the Decode map [42]. Markers that did not have an assigned position were extrapolated into the map based on physical distance to the flanking markers. Family wise multipoint LOD score curves were calculated for all autosomal chromosomes and the X chromosome using Genehunter v.2.1 [43], [44] and, in the case of family 146, SimWalk2 (v.2.91), assuming a dominant mode of inheritance. Single point LOD scores were calculated and compared to the multipoint LOD scores for data consistency. Linkages are reported according to established guidelines, that is, suggestive and significant linkage corresponding to LOD scores above 1.9 and 3.3 respectively [45]. The core linked region is defined as the region where the LOD score is larger than the maximum LOD score subtracted with 1 LOD unit (*id est* 5.5-1 = 4.5). Sensitivity analysis was performed by (i) varying the parameters in the dominant model, (ii) assessing an additive mode of inheritance, and (iii) performing nonparametric analysis. These analyses provided no further information and the results were thus omitted.

### Association analysis trios

Transmission disequilibrium test (TDT) analysis based on single SNPs was performed in Genehunter (v.2.1) and Haploview (v.3.32). p-values adjusted for multiple testing (p_adj_) were assessed by permutations between transmitted and untransmitted alleles using Haploview (v.3.32). The significance levels were also checked using the Bonferroni correction (nominal p x 66 SNPs) for consistency and no changes in significant association was found. Linkage disequilibrium calculations (D′) and plots were generated from genotype data in the trio cohort using Haploview.

### Association analysis adult case control cohort

Allelic case-control analysis based on single SNPs, comparing the allelic distribution between cases and controls in a 2×2 table, was performed in SAS (v.8.2) using Fisher's exact test. Since none of the SNPs reached nominal significance (nominal p-value below 0.05, see the Results section), no adjustment for multiple testing was performed. The analysis was also repeated comparing genotypes in a 3×2 table.

### TLESR Calculations in Dogs

Each dog served as its own control. Changes in the number of TLESRs were calculated with regard to the mean of five preceding control experiments for each dog. Data is presented as mean±SEM. Student's paired t-test was used for statistical analysis. A p-value <0.05 was regarded as statistically significant.

## Results

### Linkage analysis

**Figure 1:**
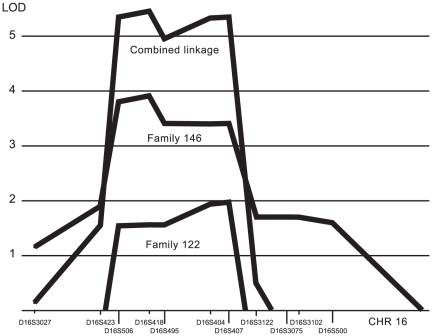
Overlapping linkage peak at chromosome 16p for families 122 and 146. Strongest linkage was obtained for family 146 showing a LOD of 3.9. The other family, 122, showed a linkage of LOD 2.0. The combined linkage curve reaches a maximal LOD score of 5.5.

Thirty-six families displaying dominant inheritance of GERD were used for whole genome microsatellite genotyping and subsequent linkage analysis. The outcome of the family wise linkage analysis was the identification of one region reaching the threshold for significant linkage and four regions reaching the threshold for suggestive linkage (Table 1). A linked region on chromosome 2 common for a subset of the families has been published previously [17]. Most of the identified linked regions were large in size due to the limited number of individuals of the families used. However, two of the families displayed an overlapping linkage to a relatively small region located on chromosome 16p. Furthermore, the linkage for one of these families was significant, reaching a maximum LOD of 3.9. The other family displayed a completely overlapping linkage curve and reached a suggestive linkage of 2.0 (Figure 1). Additional microsatellite genotyping reduced the extent of the region. Hypothesizing that the same genetic perturbation was causative in these two families, we reached a combined LOD score of 5.5 (Figure 1). The size of the region, as defined by the closest unlinked markers, common to both families, was 5.1 mega base pairs (Mbp). While the core linked region spanned only 3.6 Mbp, we were not able to reduce the linkage region any further through linkage analysis due to absence of recombination events in the core linked region.

**Table 1:** A summary of the five linked regions identified in the GERD family collection.

Families displaying genetic linkage to GERD							
Family	Number of individuals	GERD individuals	Unassigned individuals	Linked Chromosome	LOD Score	Linked region	Outer markers
101	30(14)	11(3)	7(5)	5	2.3	D5S1982-D5S424	D5S647 D5S672
103	14(8)	11(7)	0(0)	6	2.5	D6S291-D6S1570	D6S276 D6S300
122	20(10)	10(7)	5(1)	16	2.0	D16S506-D16S407	D16S423 D16S3122
146	34(14)	16(6)	6(3)	16	3.9	D16S506-D16S407	D16S423 D16S3122
103	14(8)	11(7)	0(0)	18	2.5	D18S1132-D18S1163	D18S1372 D18S464

The table shows that suggestive linkage was found on chromosome 5, 6, 16 and 18. Significant linkage was found on chromosome 16. Number of GERD individuals and individuals with unassigned disease status are presented for each family (number of males in parenthesis). The remaining individuals are healthy. The linked region is defined as the utmost markers included in the genetic linkage log of Odds (LOD) score peak while outer markers are defined as the closest genotyped unlinked marker.

### Genetic association analysis of the trio cohort

**Figure 2:**
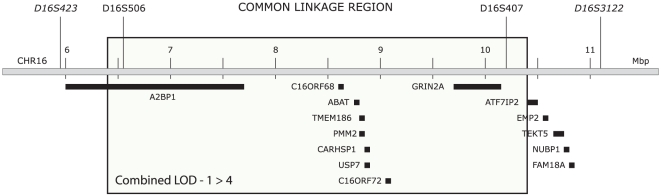
The linked region at chromosome 16p. The box represents the region of the combined maximum LOD-1. This approximates a 95% confidence interval for the location of the linked candidate gene and covers approximately 5 Mbp. The genes located within the region are; Ataxin Binding Protein 1 (A2BP1), chromosome 16 open reading frame 68 (C16ORF68), 4-aminobutyrate aminotransferase (ABAT), transmembrane protein 186 (TMEM186), phosphomannomutase 2 (PMM2), calcium regulated heat stable protein 1, 24 kDa (CARHSP1), ubiquitin specific peptidase 7 (USP7), chromosome 16 open reading frame 72 (C16ORF72) and glutamate receptor, ionotropic, N-methyl D-aspartate 2A (GRIN2A).

The identified chromosomal region defined by the combined linkage curves was relatively small and contained 9 genes (Figure 2). We argued that the causative gene most likely would be a common GERD gene, present in other GERD patients. To test this we decided to investigate if any of the genes located in the linked region were genetically associated with GERD in an independent pediatric GERD trio cohort. We selected and genotyped 66 different SNP markers located in, and around, the 9 genes. Transmission disequilibrium test analysis, followed by multiple testing adjustments for all 66 SNPs genotyped, revealed significant genetic association for the SNP rs1641021. This SNP reaches an adjusted p-value of 0.027 (calculated as the nominal p-value 0.0004 multiplied with 66). The data for this SNP, together with the 16 other SNPs located in the ABAT region, are shown in table 2. The associated SNP, rs1641021, is located within intron 16 of ABAT.

**Table 2:** Genetic association in the trio cohort.

Transmission disequilibrium test analysis for the genotyped SNPs located in the ABAT gene region of chromosome 16										
		All Individuals n = 219			Endoscopy positive n = 177			Histology positive n = 175		
#	SNP	T:U	p-val	p_adj_	T:U	p-val	p_adj_	T:U	p-val	p_adj_
3	rs2302607	61:61	1	1	55:49	0.56	1	49:48	0.92	1
4	rs1273350	90:65	0.045	0.91	71:50	0.056	0.96	77:50	0.017	0.60
5	rs6497327	97:81	0.23	1	79:67	0.32	1	81:68	0.29	1
6	rs1940989	89:83	0.65	1	76:67	0.45	1	70:66	0.73	1
7	rs1731017	88:85	0.82	1	74:71	0.80	1	68:68	1	1
8	rs1273398	29:26	0.69	1	22:21	0.88	1	25:22	0.66	1
**9**	rs1641021*	**122:73**	**0.0004**	**0.027**	**100:57**	**0.0006**	**0.037**	**100:54**	**0.0002**	**0.012**
10	rs1079348*	112:86	0.065	0.97	96:67	0.023	0.72	90:67	0.066	0.97
11	rs3743798*	112:102	0.49	1	96:85	0.41	1	88:79	0.49	1
12	rs2304471*	80:60	0.091	0.99	68:48	0.063	0.97	64:44	0.054	0.94
13	rs67497537*	100:71	0.027	0.77	89:57	0.0081	0.37	81:51	0.0090	0.39
14	rs28595449*	83:56	0.022	0.69	72:45	0.013	0.51	69:40	0.0055	0.26
15	rs2075827*	90:62	0.023	0.72	77:50	0.017	0.62	74:45	0.0079	0.35
16	rs1657070*	99:87	0.38	1	84:68	0.19	1	79:69	0.41	1
17	rs8097	98:98	1	1	83:81	0.88	1	80:80	1	1
18	rs2304446	72:60	0.30	1	57:50	0.50	1	58:48	0.38	1
19	rs30764	73:71	0.87	1	62:53	0.40	1	61:59	0.86	1

A total of 66 SNPs were genotyped in the linked region on chromosome 16 and subsequently analyzed with TDT. The table shows a subset of these located around ABAT. T:U denotes the number of transmitted versus untransmitted alleles. p-val is the nominal p value. p_adj_ is the p-value adjusted for multiple testing through permutation analysis for the full 66 SNP panel. The trio cohort consists of 177 probands with positive findings from endoscopy evaluation and 175 probands with positive findings from histology evaluation. In total the cohort consist of 219 trios. The eight SNPs marked with an asterisk were also genotyped in the adult case control cohort in addition to rs8046201.

**Figure 3:**
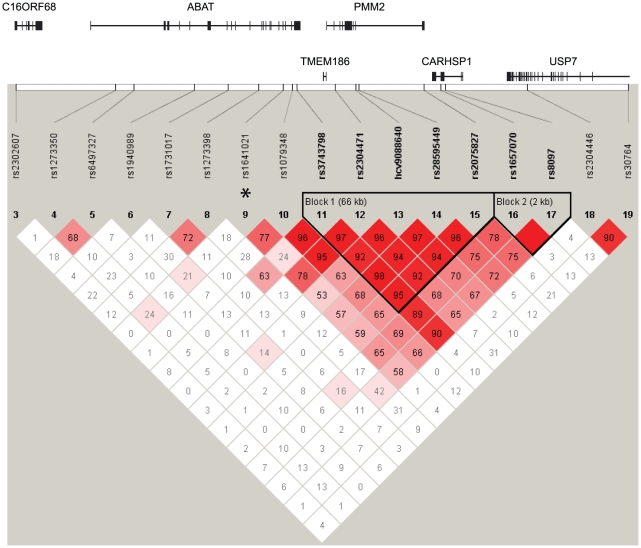
LD map of the region containing ABAT. The associated SNP, rs1641021 is located adjacent to a LD block covering the 3′ part of ABAT together with TMEM186, PMM2 and CARHSP1.

The genetic association we identified for rs1641021 could originate from ABAT, or depending on the Linkage Disequilibrium (LD) properties of the region, from a gene located nearby. We therefore constructed a LD map from the trios used in this study (Figure 3). This map shows that rs1641021 lies outside an LD block which covers the 3′ end of ABAT together with the neighboring genes TMEM186, PMM2 and CARHSP1. The LD structure, surrounding the associated SNP rs1641021, implies that this marker is linked with a nearby located true disease associated mutation. We therefore conclude that the association seen for rs1641021 originates from ABAT.

### Genetic association analysis of the adult case control cohort

In an effort to replicate the association in the trio cohort, nine SNPs distributed over the ABAT LD block were genotyped in the adult case control cohort (Table 2). The associated SNP in the trio cohort (rs1641021) reached a nominal p = 0.34 (nominal p value between 0.24 and 0.94 for the other SNPs). None of the genotyped SNPs showed an indication for association. The allele frequency of rs1641021 did not differ between the trio cohort (41%), the controls in the case-control cohort (45%) or the frequency published for Caucasians in the Hapmap project (42.4%) [46]. The statistical power was calculated in this cohort by estimating the risk ratio in trios for the minor versus major allele to 1.67 (122/73). Assuming a minor allele frequency of 0.4 in controls, a risk ratio of 1.5, and that 250 cases and 475 controls have been successfully genotyped, the power to replicate the rs1641021 association in the adult case-control cohort was 95%. The power was 80% to detect an association at the level of significance 0.0055 (Bonferroni correction for 9 SNPs).

### Sequencing

We subsequently sequenced the entire coding sequence of ABAT, including intron-exon splicing boundaries, in three affected and three healthy individuals from each of the two families showing linkage to chromosome 16p. No amino acid changing mutation was identified.

### TLESR measurements in dogs

**Figure 4:**
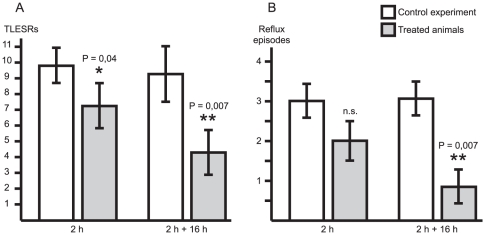
Analysis of ABAT inhibition in dogs. Inhibition of TLESRs (A) and reflux episodes (B) in dogs after administration of the ABAT specific inhibitor Vigabatrin. Administration was performed 2 hours or 2 and 16 hours before measurements. Each group consisted of six dogs. TLESR and reflux events are presented as number of events/45 minutes. P-values were calculated using paired t-test. Open boxes represent the control experiment, filled boxes are after the administration of Vigabatrin, n.s. denotes not significant.

In an attempt to better understand the role of ABAT in GERD, we assessed the effect of vigabatrin, a selective irreversible inhibitor of ABAT, on TLESRs in dogs. A single dose of vigabatrin, administered 2 h (100 mg/kg) before the start of the experiments, produced a statistically significant inhibition of TLESRs by 30%±9.8%. When vigabatrin (100 mg/kg) was administered twice (at 2 h and 16 h) before the experiment, the inhibitory effect was further increased to 57.3%±11.4% (Figure 4A). The average number of TLESRs in all control experiments was 9.9±0.5. In addition, vigabatrin produced a statistically significant reduction of the number of acid reflux episodes from 3.1±0.4 (control) to 0.8±0.4 (100 mg/kg dosed 2 h and 16 h) during the 45 min measurement period. A single dose of vigabatrin produced no significant reduction of reflux episodes (3±0.4 and 2±0.5 in control and vigabatrin treated animals, respectively) (Figure 4B).

## Discussion

We have investigated the genetic predisposition to GERD using three different patient cohorts. We started by performing a linkage analysis in a collection of 36 families with the aim to identify both general and family specific linked chromosomal regions. These families were selected for the presence of GERD in every generation, in a dominant fashion, to maximize the genetic signal. We analyzed the linkage data both collectively (as previously published in [17]) and familywise and it became clear to us from this analysis, where we demonstrate linkage to five different chromosomal regions, that GERD is a disease with many different genetic predispositions.

In the remainder of this work we focused on the two families that showed linkage to the same region on chromosome 16. These linkages were identical to the resolution provided by microsatellite mapping and although the region was relatively small, it contained nine genes.

To investigate if any of these nine genes were genetically associated with GERD we used a separate patient collection with sufficient resolution to allow mapping of single genes. We genotyped 66 SNPs distributed over the nine genes in a pediatric GERD trio cohort. Applying multiple testing adjustments using permutations analysis we identified a significant association in ABAT, but not in any of the other genes, showing that ABAT is a GERD associated gene in our pediatric GERD population. The cohort consists of more males than females but we cannot detect a gender bias in the association. We attempted to replicate the genetic association in ABAT using an adult Swedish GERD case-control cohort by testing the associated ABAT SNP together with surrounding SNPs but found no evidence for genetic association. Statistical power estimates do not explain the lack of replication in this cohort.

This absence of association may be due to the fact that the proband in the Australian families were pediatric cases, the trio cohort is also pediatric and collected in Australia but the Swedish case-control cohort consists of adult GERD cases. Furthermore, the population history clearly differs between the two cohorts. The adult case-control cohort is a mixed Swedish and Finnish material with Saami influence while the trio cohort is mainly Anglo-Celtic. In our previous publication, we demonstrated that COL3A1 alleles were reciprocally associated with GERD (see table 2 in [17]) implicating that GERD mutations have arisen separately in these two different patient cohorts. Our conclusion is that ABAT alleles are not present as genetic risk factors for GERD in the Swedish adult case control cohort. However, the statistically significant association in the trio cohort clearly shows that one polymorphic variant within ABAT is associated to GERD. From our sequencing results we demonstrate that the open reading frame of the ABAT gene is intact. The few known disease causing mutations in the ABAT protein cause psychomotor retardation and early death [47], [48]. Perhaps severe mutations are not to be expected in a disease such as GERD. More likely, a GERD associated mutation in ABAT may be expected to be located in non-coding sequences such as regulatory elements or non coding RNAs, as has recently been discussed in the literature regarding complex diseases [49].

Due to ABATs predominant expression in neuronal tissues it was not possible to investigate the function of ABAT in GERD patients because suitable samples were unobtainable to us. Instead, we investigated how ABAT perturbation may lead to the development of GERD through inhibition of ABAT function *in vivo* in dogs. Administration of the ABAT specific inhibitor vigabatrin significantly inhibited TLESRs and reflux episodes in dogs. This result implies that humans carrying disease associated ABAT alleles may suffer from an increased number of reflux episodes due to dysregulation of the LES.

ABAT catalyses the first step in the degradation of GABA. Inhibition of ABAT leads to elevated levels of GABA in the synaptic junctions causing increased GABA-mediated signaling through the GABA receptors [50]. The neurotransmitter GABA is involved in the control of the LES [51]. Signaling through both GABA_A_ [52] and especially GABA_B_ receptors reduces TLESRs, as shown with GABA_B_ receptor agonists [53]. TLESRs are the major motility factor underlying GERD [54] and these relaxations result in an increased frequency of reflux episodes [55] thereby causing esophageal mucosal damage.

The previously published association between COL3A1 and GERD [17] represents a disease mechanism presumably involving tissue vulnerability of the esophagus. The ABAT association we present here indicates that GERD in addition has a neuronal disease component.

Our results leading to the identification of ABAT are in line with the ongoing efforts to develop antireflux drugs to treat GERD. These drugs are GABA analogs specifically directed against the GABA_B_ receptor [53]. Our findings support the involvement of GABA with GERD. This raises the question of whether other GABA signaling components such as transporters and receptors are associated with GERD as well.

In conclusion, we show that a polymorphic variant within the ABAT gene is associated with GERD and that ABAT inhibition leads to a reduction of TLESRs and acid reflux events. Our results raise the possibility of treating GERD by inhibiting ABAT function.
